# Evaluating the Quality of Website Information of Private-Practice Clinics Offering Cell Therapies in Japan

**DOI:** 10.2196/ijmr.5479

**Published:** 2016-05-24

**Authors:** Hidenori Kashihara, Takeo Nakayama, Taichi Hatta, Naomi Takahashi, Misao Fujita

**Affiliations:** ^1^ Center for iPS Cell Research and Application (CiRA) Kyôto University Kyôto Japan; ^2^ Graduate School of Public Health Department of Health Informatics Kyôto University Kyôto Japan

**Keywords:** stem cell, health information, regulations, regenerative medicine, misrepresentation, medical tourism, direct-to-consumer, online marketing, web survey, descriptive analysis

## Abstract

**Background:**

Although the safety and effectiveness of stem cell therapies are yet to be proven, recent studies show that such therapies are being advertised with some questionable marketing techniques to effect positive portrayal of the therapies on the webpages of private-practice clinics to sell their therapies worldwide. In such context, those clinics communicate directly with consumers (patients and their family members) via the clinics’ websites. Meanwhile, the Health Science Council at the Ministry of Health, Labour, and Welfare (MHLW) in Japan has pointed out noncompliance of some local clinics with the provisions concerning medical advertising in the Medical Care Act in the past. However, locally little is known about the current status of those clinics including the quality of their webpage information disseminated.

**Objective:**

To evaluate the quality of website information of private-practice clinics offering cell therapies in Japan.

**Methods:**

Twenty-four websites with 77 treatments from the Google search were identified for evaluation. The following three exploratory analyses were performed: first in order to ascertain web-based portrayal of private-practice clinics offering cell therapies, a descriptive analysis was conducted using a coding frame; second we evaluated the quality of the target website information from the viewpoint of the level of consideration taken for patients and their family members, using 10 quality criteria (“the Minimum Standard”) from the e-Health Code of Ethics 2.0; third we counted and coded expressions that matched set categories for “name-dropping” and “personalized medicine” in the information posted on these websites.

**Results:**

Analysis on the treatments (N=77) revealed 126 indications (multiple response): the top three indications were “cancer,” “skin-rejuvenation/antiaging/anti–skin aging,” and “breast augmentation/buttock augmentation.” As for the portrayal of treatment risks and benefits, 78% (60/77) of treatments were mentioned with “benefits,” whereas 77% (59/77) of treatments were mentioned with “risks.” As for the source(s) cited for the discussions of treatment risks and benefits, no treatment quoted an expert’s opinion for the risks, whereas 7% (6/77) treatments quoted external sources for the benefits. As for the results with e-Health Code of Ethics 2.0, not a single clinic fulfilled all the 10 criteria; 63% (15/24) of the clinics was found exercising “name-dropping,” and 21% (5/24) of the clinics mentioned expressions related to “personalized medicine” on their websites.

**Conclusions:**

Our website content analyses confirmed the following: (1) the clinics mentioned the risks or benefits of the treatments with hardly any scientific citations, (2) the way the website information was disseminated was inappropriate for patients and their families, and (3) many websites seemed to be using marketing techniques in order to draw patients’ interests or attentions. It is important that more similar studies are undertaken globally to enable an orchestrated regulatory approach toward private-practice clinics.

## Introduction

The use of regenerative medicine, including stem cell therapy, has been experimentally attempted to treat diseases that cannot be cured by conventional treatment methods [1]. Currently, little is known about the safety, efficacy, or effectiveness of regenerative medicine. Thus, as a rule, experts do not recommend the use of this technology for the treatment of patients outside the framework of research [2]. Meanwhile, in reality patients from many countries are accessing website information related to unapproved regenerative therapies, crossing borders if necessary to visit private-practice clinics that offer such therapies for hefty fees [3]. In such context, the Internet plays an important role, and in fact, those private-practice clinics communicate directly with consumers (patients and their family members) via the Internet [4,5].

However, recent studies have raised some questions about problematic ways that website information is being advertised with some marketing techniques [6,7]. For example, one previous study indicated that there were websites that used several marketing methods related to information concerning stem cell treatments in order to affect a positive portrayal of the treatments [6]. The market trend to use the language of research papers, including those concerning stem cell research, has also been indicated in cases when the stem cell business industry advertises their own antiaging stem cell products via the Internet [7]. In addition, there is a common advertising technique known as “name-dropping,” which is defined by a web-based dictionary [8] as follows: “The act of talking about famous people that you have met, often pretending that you know them better than you really do, in order to appear more important and special.” In response to such advertising, the International Society for Stem Cell Research expressed concerns in its handbook that patients and their family members should not be rushed to make a decision on receiving treatment based on the web-based information disseminated by these clinics [9].

In Japan, an incident in which a Korean patient who received stem cell therapy died of pulmonary embolism was reported in 2010 [10]. At that time, there were no laws in Japan concerning regenerative medical treatments that were not covered by public health insurance. However, after the enforcement of the 2014 Act on the Safety of Regenerative Medicine (ASRM), such clinics became bound by the same regulations as clinical research [11]. A medical institution that attempts to offer regenerative medical treatments not covered by health insurance (ie, private practice) must first submit a plan to a Certified Special Committee or a Certified Committee, and after receiving an approval from said committee, submit the approved plan to the Ministry of Health, Labour and Welfare (MHLW) [11].

Now, new laws and regulations are in place, but the current situation of private-practice clinics offering cell therapies in Japan remains unclear. This remains so in spite of the fact that some private-practice clinics offering cell therapies in Japan have been indicated to have a variety of problems [11]. For example, before the enforcement of the ASRM, the Health Science Council of the MHLW was informed that some private-practice clinics in Japan offering cell therapies were in violation of the regulations concerning advertising that are stipulated in the Medical Care Act [12]. However, the actual status of these violations have not been elucidated either.

An assessment of the current problems and concerns of regenerative medicine as referred to earlier reveals the following points: (1) although the use of various marketing techniques such as name-dropping on English language websites has been investigated, the current status of Japanese language websites remains unclear, and (2) it has been indicated that some Japanese private-practice clinics offering cell therapies treatments use medical advertisements that are in violation of the regulations concerning advertisements stipulated in the Medical Care Act [12], but the current status of the problem is unclear.

In order to ascertain the current situation surrounding private-practice clinics offering cell therapies in Japan, we evaluated the quality of the clinics’ website information from consumers’ points of view.

## Methods

### Analysis Overview

In the present study, after systematically downloading the websites of these clinics, we performed the following three exploratory analyses. Analysis 1: in order to ascertain the portrayal of private-practice clinics offering cell therapies, we performed a descriptive analysis. Analysis 2: we evaluated the information of these websites concerning regenerative medicine from the viewpoint of the level of consideration taken for patients and their family members, and if that information was appropriately disseminated. Analysis 3: we counted and coded expressions that can be construed as name-dropping in the information posted on these websites. The present study is a part of the grant-aided project to investigate the status of regenerative medical treatments in Japan (see Acknowledgements), thus part of the data is shared with a previous study [13].

The definitions for regenerative medicine in the present study are based on the definitions found in the ASRM [11]. For more detailed definitions of the terms, please see the Multimedia Appendix 1 (Scope of Application of the ASRM).

### Data Collection

The results of a multi-stage extraction of the websites are as follows:

1. Google search: our study is based on a sample of websites identified from May 24, 2014 to May 27 using Google search engine and the browser Mozilla Firefox with the following keywords: (“cell therapy” OR “regenerative medicine” OR “stem cells”) AND {(“private practice” OR “private expenses”) OR (“hospital” OR “clinic” OR “medical office” OR “doctor’s office”)}. The language of the actual search terms used was Japanese. We used the following search expressions: (“saibo chiryo” OR “saisei iryo” OR “kansaibo”) AND {(“jiyu shinryo” OR “jihi”) OR (“byoin” OR “kurinikku” OR “shinryosho” OR “iin”)}. The results of the search showed approximately 1,590,000 hits, of which 836 could be displayed.

2. Storage of data: of the 836 hits, 762 hits (clinics that do not provide regenerative medicine stipulated by ASRM, public research institutions/public health institutions and university hospitals, program information, such as television, news, and blogs, hospital information sites, and duplicate sites) were excluded. The remaining 74 websites were electronically saved using Firefox add-on software ScrapBook 1.5.9.

3. Selection of the targets for analysis: based on the following two reasons, the present study was limited to analysis of 24 websites; it has been indicated that the top 20 websites displayed on the browser reflect the actual websites that search engine users view [14,15]. Moreover, the principal aim of the present study was to perform an exploratory investigation.

### Analysis 1: Descriptive Analysis

In order to ascertain the overview of the 24 clinics that were extracted above, we performed a descriptive analysis of the information concerning the clinics themselves and treatment methods listed on the websites of the clinics. We analyzed (1) clinic location/foreign language page and (2) advertised departmental name. With regard to information concerning treatment methods, of the 24 clinics that offer therapies, we collected 77 regenerative medical treatments that fell within the scope of the ASRM and analyzed the following items: (3) target disease, (4) method of transplantation (“local,” “general,” “unknown/not listed”), (5) the presence or absence of explanation of benefits and effects (Yes/No), (6) cited evidence of benefits or efficacy (if any) from scientific journals, (7) the presence or absence of description of risks and safety (Yes/No), (8) cited evidence of risks or safety (if any) from scientific journals, and (9) costs (ie, cost of each therapy or treatment if mentioned).

Moreover, with regard the items concerning the benefits and risks of treatment (items 5-8, above), we created a coding frame while referring to a previous study [15]. Websites in which a reference to the benefits and efficacy of treatments (eg, “the effects are not the same in all patients,” “your skin will appear 10 years younger,” and “the risks are different for each person”) could be confirmed were evaluated as “Yes” with regard to explanation of benefits and efficacy. Descriptions that clearly listed information concerning safety and expected side effects of said treatments (eg, “It is safe because samples are cultured in a clean cell processing center” and “A fever may occur after infusion of immune cells”) were evaluated as “Yes” with regard to explanation of risk and safety. On the other hand, descriptions that listed only basic medical explanations such as the characteristics of the cells used in treatment and their mechanisms of action were evaluated as “No.” In items 6 and 8, we confirmed the presence of research citations, which served as the basis for claims of benefits and risks made on the websites. Data confirming process for items 1 to 6 was already implemented by two authors (TH and MF) in the previous study [13], and as for 7 to 9 the coding validity was confirmed by two authors (HK and TH).

### Analysis 2: Assessment with e-Health Code of Ethics 2.0

The “e-Health Code of Ethics 2.0” was used in order to evaluate the quality of website information to ascertain whether said information was appropriately provided for patients and their family members who need information concerning regenerative medicine [16]. The Code was created by the nonprofit organization Japan Internet Medical Association (JIMA), and its origin can be traced back to the “Medical Information Usage Guidebook” developed in December 1999 [17].

The following 10 quality criteria called the “Minimum Standards” from the e-Health Code of Ethics 2.0 were used in our study. See Multimedia Appendix 2 (The 10 Quality Criteria of Minimum Standard from e-Health Code of Ethics 2.0) for more details. We compared the information presented on the 24 websites that were targets of the present study with the 10 Minimum Standards using the following three scales: “1- Complied,” “2- Not complied,” “3- Not Applicable (NA).” The outline of the 10 Standards is as follows:

1. Disclosure of information about the website operator

2. Disclosure of information about sponsorship

3. Provision of contact center for further inquiries

4. Clarifying intended recipients of content

5. Disclosure of the information concerning writing, production, and/or editorial supervision of conduct

6. Adherence to relevant laws and regulations

6-1. Prohibited advertising of names of the medical institutions (eg, “Regenerative Medicine Clinic”)

6-2. Prohibited advertising of names of hospital departments (eg, “Department of Regenerative Medicine”)

6-3. Prohibited claim of specialization (eg, “Certified Specialist in Regenerative Medicine”)

6-4. Prohibited use of the term (eg, “regenerative medicine”) in the explanations of treatments

6-5. Prohibited use of photos (eg, claiming the effectiveness of a surgery by showing the pre- and post-operational photographs of patients)

7. Notification to the users of profit-oriented activities on the websites

8. Displaying a pop-up message box that reminds the user that they were being transferred to external websites when clicking the links to external websites

9. Displaying handling of personal information

10. Displaying a privacy protection policy

One author (HK) evaluated 24 websites based on the 10 Standards, and another author (TH) verified the coherence of the evaluation. To keep the reliability of evaluation, two authors jointly went back to each website and referred to the relevant section of e-Health Code of Ethics 2.0.

### Analysis 3: Focusing on “Name-dropping”

We counted clinics that used the following types of items that fell under the category of expressions of name-dropping on the homepage of their respective websites: academic conference presentation, articles published in academic journals, media coverage, anecdotes by celebrities, medical doctors or specialists, and governmental or regulatory authorities/academic institutions.

Moreover, techniques similar to name-dropping include the use of language used in research papers in order to show the plausibility of the treatment method [7]. Personalized health care has been proposed in an integrated conceptual model where not only genomic medicine but also advanced technologies such as regenerative medicine are included as components [18]. In the present study, we focused on language similar to “personalized medicine,” and counted the number of clinics using the following expressions: “Order-Made Medicine,” “Tailor-Made Medicine,” and “Personalized Medicine.”

Two authors (HK and TH) independently evaluated 24 websites. When there was a disagreement in evaluation results, the two authors jointly went back to each website in question and discussed the issues until all disagreements were resolved.

## Results

### Descriptive Analysis

The results of the descriptive analysis of information concerning the 24 target clinics and information concerning 77 treatments are mentioned below. As for the locations of clinics and presence of foreign language websites, the majority of clinics were concentrated in the capital of Tokyo and 21% (5/24) of the clinics had a foreign language website. For more details of the clinics’ demographic information, see Multimedia Appendix 3. Concerning advertised departmental names, a wide variety of 33 departmental names were observed in 19 clinics, but five clinics did not specifically advertise any departmental names. The type of clinic with the highest number was cosmetic dermatology followed by cosmetic surgery, internal medicine, and dentistry (Textbox 1). Please note that the numbers in parenthesis in the Textbox are the subtotal of each departmental name(s) used by 24 clinics.

**Departmental Names Used by 24 Clinics (multiple response).**

Aesthetic Dermatology (7) Plastic Surgery (5) Internal Medicine (3) Dentistry (3) Immunotherapy (2) Aesthetic Medicine (2) Plastic Surgery (2) Regenerative Medicine (2) Aesthetic Dentistry (2) Cancer Immunotherapy (1) Regenerative Medicine of Skin (1) Neurosurgery (1) Neurology (1) Radiology (1) Orthopaedics (1) Cardiology (1) Urology (1) Surgery (1) Gastrointestinal Medicine (1) Proctology (1) Dermatology (1) Medical Oncology and Immunology (1) Medical Oncology (1) Mammary Gland Medicine (1) Gynaecology (1) Respiratory Medicine (1) Obstetrics (1) Paediatrics (1) Dental Surgery (1) Orthodontic Dentistry (1) Preventive Dentistry (1) Haematology (1) Paediatric Dentistry (1) Unknown (5)

### Information Concerning Treatment Methods

With regard to target diseases, the authors observed 126 target diseases and symptoms (nondisease). This is shown in Table 1.

With regard to “transplantation methods,“ local injection was the most commonly performed method (30 treatments). Twenty-five treatments (approximately one-third of all treatments) fell under the category of ”unknown/no description” (Table 2).

Table 3 shows that 78% (60/77) of the treatments listed the expected benefits and efficacy, and Table 4 indicates that 77% (59/77) of the treatments listed information concerning safety and expected side effects. Six citations from scientific papers concerning the benefits and efficacy of the treatments were confirmed, but no citations concerning the safety and side effects of treatments were noted. Moreover, all of the cited studies supported the benefits and efficacy of the treatment method in question, and no literature that cast doubt on the treatment efficacy was cited. With regard to costs, the prices were clearly listed for 82% (63/77) of the medical treatments, and 18% (14/77) of the treatments had no information concerning prices.

**Table 1:** Stated indications of treatments (multiple response) (N=77).

**Diseases** ^a^			n
		Cancer	42
		Diabetes/type I diabetes	6
		Myocardial infarction	4
		Cerebral infarction	4
		Hepatitis	3
		Renal failure	3
		Rheumatoid arthritis	3
		Alveolar bone atrophy/missing jaw bone/missing teeth	3
		Alopecia	3
		Cirrhosis	2
		Refractory ulcer	2
		Periodontosis	2
		Collagenosis	2
		Osteoarthrosis	2
		Vascular dementia	2
		Parkinson’s disease	2
		Immunological diseases	2
		Burger disease	2
		Liver diseases	2
		Lower limbs ischemia/critical limb ischaemia/peripheral artery diseases	2
		Atopic dermatitis	1
**Nondiseases** ^a^		
	Skin beauty/antiaging		19
	Breast augmentation/buttock augmentation		7
	Nutritional fortification/immunostimulation		2
	The glans/penis enlargement		1
	Nontypable		3
**Total** ^b^			126

^a^Indications have been classified into “Diseases,” which are based on the International Statistical Classification of Disease and Rerated Health Problems, version 10 (ICD-10) [19], and into “Nondiseases,” which is based on the patients’ own symptoms and conditions, and accordingly labelled.

^b^The frequencies do not sum to n=77 because several treatments fitted into more than one category per concept.

**Table 2:** Route of administration of treatments (N=77).

Route of administration		n	%
	Local	30	39
	Systemic	18	23
	Local and Systemic	4	5
	Unknown/Not specified	25	33
**Total**		77	100

**Table 3:** Mentioned benefits for treatments (N=77).

Account of benefits		n	%
**Applicable**		60	78
	Scientists or researchers	5	7
	Medical specialists	0	0
	Others	1	1
	No citations from the third party	54	70
**Not applicable**		17	22

**Table 4:** Mentioned risks for treatments (N=77).

Account of risks		n	%
**Applicable**		59	77
	Scientists or researchers	0	0
	Medical specialists	0	0
	Others	0	0
	No citations from the third party	59	77
**Not applicable**		18	23

### Assessment with e-Health Code of Ethics 2.0

**Figure 1:**
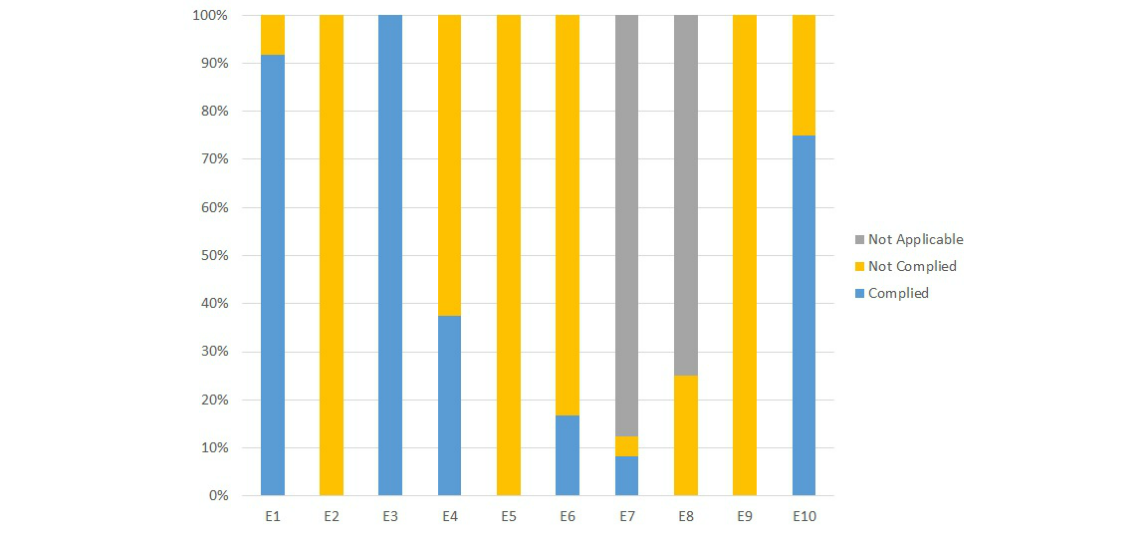
Reviewed Websites (n=24) and their Compliance with e-health Code of Ethics 2.0. E1 Disclosure of information about the website operator; E2 Disclosure of information about sponsorship; E3 Provision of contact center for further enquiries; E4 Clarifying intended recipients of content; E5 Disclosure of the information concerning writing, production, and/or editorial supervision of conduct; E6 Adherence to relevant laws and regulations; E7 Notification to the users of profit-oriented activities on the websites; E8 Displaying a pop-up message box that reminds the user of being transferred to external websites when clicking the links to external websites; E9 Displaying the handling of personal information; E10 Displaying a privacy protection policy.

Of the websites of the 24 clinics that were a target of study, no website was compliant with all of the 10 Minimum Standards of the e-Health Code of Ethics (Figure 1). The items with the highest compliance rates were E3 (provision of contact center for further enquiries) (100%, 24/24), followed by E1 (disclosure of information about the website operator) (92%, 22/24), and E10 (displaying a privacy protection policy or privacy policy) (75%, 18/24). The three items with the lowest compliance rates (0%, 0/24) were as follows: E2 (disclosure of information about sponsorship), E5 (disclosure of the information concerning writing, production, and/or editorial supervision of conduct), and E9 (displaying handling of personal information).

We conducted an evaluation of violations of related laws and regulations stipulated in E6 (adherence to relevant laws and regulations) using a five-point negative list. A negative list approach requires listing those items that are prohibited as exceptions while those not listed in the list are in principle deemed permitted. The most common item of the negative list was E6-4 “prohibited use of the term (ie, regenerative medicine) in the explanations of treatments“ on the websites (71%, 17/24). The second most common item was E6-5 ”prohibited use of photos” (ie, claiming the effectiveness of a surgery by showing the pre- and post-operational photographs) (54%, 13/24). With regard to item E6-2 “prohibited advertising of names of hospital departments,” 37% of the clinics (9/24) advertised the name “regenerative medicine department” (Textbox 1). The least common item was E6-3 “prohibited claim of specialization” (ie, certified specialist of regenerative medicine), and no sites using this expression in relation to specialty was confirmed. Following this, one clinic 1% of the clinics (1/24) fell under item E6-1 “prohibited advertising of names of the medical institutions” by using the term “Regenerative Medicine Center” or “Regenerative Medicine Clinic.”

### Focusing on Name-Dropping

Expressions related to name-dropping were observed on 63% (15/24) of websites (Table 5), and of these, expressions related to media coverage were the most common (n=10). For example, the names of television or radio programs that covered the clinic were listed on the homepage of websites. Moreover, with regard to governmental or regulatory authorities/academic institutions or associations, five websites using the names of universities that have conducted clinical research in collaboration with the clinic were confirmed, but use of the names of Japanese or international regulatory authorities or organizations associated with patent sales was not confirmed.

The use of the term “personalized medicine” or similar language was confirmed in 21% (5/24) of sites. Of these, the expression “Personalized Medicine” was observed in one site and “Order-Made Medicine” in four.

**Table 5:** Name-dropping and citations on clinics’ websites (N=24).

Codes		No. of clinics
**Applicable (name-dropping)** ^a^		15
	Academic conference presentations	2
	Articles published in academic journals	2
	Media coverage^b^	10
	Anecdotes by the celebrities^c^	4
	Medical doctors or specialists^d^	4
	Regulatory authorities/academic institutions or associations^e^	5
	Others	0
**Not applicable**		9

^a^In total, 15 clinics were found to be name-dropping. However, frequencies do not sum to n=15 as several clinics referred to more than one category per citation.

^b^Examples include TV programs or beauty magazines.

^c^Examples include the head of a well-known beauty clinic in Japan, a female celebrity, and a famous scientist.

^d^In this category, an individual with either MD or PhD title as a specialist was counted.

^e^Examples include regulatory authorities or academic associations as well as universities that are associated with the clinic in question for the purpose of joint research projects.

## Discussion

Principal Findings

The present study is the first study to analyze the quality of information presented on the websites of Japanese private-practice clinics offering cell therapies from the viewpoint of patients and their family members. The results of the present study revealed that in total, 24 clinics specializing a wide variety of specialties offered 77 treatment methods for a variety diseases and conditions. No clinics complied with the 10 Minimum Standards of the e-Health Code of Ethics 2.0, which was developed as a guideline for medical institutions to create websites with high transparency. Moreover, approximately two-thirds of clinics used name-dropping on the home page of the website. Here, we discuss several noteworthy results of the present study.

### Status of Adherence to the e-Health Ethical Code

The JIMA, the developer of the e-Health Code of Ethics 2.0, defines e-Health as “the continued provision and use of highly valuable medical information in the fields of medicine and insurance, with the assistance of new information and communication technologies such as the Internet” [20]. This suggests that the information listed on the websites of the target clinics offering cell therapies was not of high value from the viewpoint of patients and family members. It is desirable that clinics that offer such therapies are committed to careful dissemination of information to patients and their family members.

Upon viewing each item of the e-Health Code of Ethics 2.0, it should be noted that almost no clinics adhered to the items of “E2 Disclosure of information about sponsorship,” “E5 Disclosure of the information concerning writing, production, and/or editorial supervision of conduct,” or “E9 Displaying the handling of personal information.” If information concerning sponsorship and the author of the contents of the medical information is not displayed, it is difficult for patients and their family members to judge the information on websites concerning conflict of interests and attribution, and the following points can be suspected: (1) the possibility that the contents and data are favorably biased toward the managing operators and sponsors, (2) the possibility that fair information is not objectively provided without being influenced by the sponsor, and (3) the possibility that the information provided on the website is not based on the appropriate judgment of a medical specialist. Considering the fact that the 24 websites that were a target of the present study contained no information concerning the handling of personal information, doubts remain whether website operators have appropriately maintained confidentiality or not.

When creating the criteria to assess E6 (adherence to relevant laws and regulations), the law concerning medical advertisements (the Medical Care Act) was referred to, but several supplementary explanations are necessary to interpret these results. The Medical Care Act that governs medical advertising issued by medical institutions such as flyers, newspaper advertisements, and advertisements in print media, does not cover websites. Although the Japanese MHLW has issued guidelines concerning the advertisements of websites, these have no legal force [21]. Accordingly, the following conclusion is reached: if the extent of the current law were extended to regulate websites, more than 80% of the private-practice clinics offering cell therapies on the present study would be subject to prosecution.

### Name-Dropping and Scienceploitation

Expressions that fell under name-dropping were used by two-thirds of the 24 clinics that were targets of the present study. Many websites that posted information designed to capture the interest of patients and their family members on the respective homepages were confirmed (Table 5). However, almost no websites that cited a scientific basis for treatment could be confirmed (Tables 3,4). The same trend has been reportedly observed in the websites of beauty/health websites, nonstem cell–based cosmetic companies, web-based news sources, stem cell–based cosmetic companies, web-based magazines, beauty/health blogs, and stem cell supplement companies [7]. Based on the results of such previous studies, it can be inferred that the targeted transmitters of the websites information also believe that name-dropping actions such as displaying news reports, photographs, and comments from celebrities, have equivalent or greater effects in eliciting the interest of patients and their family members, in comparison with displaying the scientific basis for treatment.

Petersen et al [6] focused on marketing techniques referred to as “representational devices,” that is, the websites of said clinics that emphasize having a human network of experts and cooperative relationships. Moreover, direct-to-consumer advertising that relies on claims that appear to be scientific to wrongfully attract patients and their family members through media such as websites is called “misrepresentation” [22]. The industry trend for excessively inappropriate misrepresentation based on the relentless pursuit of profit is referred to as “scienceploitation” [23], a phenomenon that has been widely observed in fields related to regenerative medicine [7].

However, not all name-dropping and “representational devices” are necessarily ethically inappropriate. For example, in the present study, we counted some listing of names of universities as name-dropping, but most cases were clinical research conducted jointly with the clinics. It is difficult to say that such information itself unfairly exploits patients and their family members. Thus, the expressions that fell under the category of name-dropping in the present study contained a mixture of some problematic expressions that could be construed as scienceploitation and others that were not. If we assume that the phenomenon known as scienceploitation can be empirically elucidated, then further research by having actual users such as patients and their family members browse the website and asking their impressions and opinions would be necessary.

### The Need for Global Criteria

In the present study, we adopted the 10 Minimum Standards of the e-Health Code of Ethics 2.0, but these are primarily the quality criteria for evaluating overall forms of websites, and not necessarily best fit to evaluate the contents of the websites. However, the contents of the information provided on websites is important information for patients to make a decision whether to actually receive treatment. For this reason, in the present study, we created items related to risks and benefits as well as name-dropping and subsequently conducted analyses of the items so that we could evaluate this point. The results of the present study revealed that there is a problem in the quality of information: many websites do not cite any scientific basis for their claims and tend to use name-dropping in order to attract the attention of patients. Using the Minimum Standards of the e-Health Code of Ethics 2.0 alone would not have revealed such findings.

Interestingly, the full version of the e-Health Code of Ethics 2.0 contains the following phrase: “in cases when the evaluation of the provided information cannot be determined, consideration toward users should be made by adding an explanation or providing reference information so that users can judge for themselves” [16]. This type of item that evaluates the contents of information is particular to the e-Health Code of Ethics 2.0, and has not been confirmed in other similar Japanese or international guidelines [24-27]. Such criteria are deemed essential when evaluating the websites of private-practice clinics providing unestablished cell therapies with information of questionable quality. If the said criteria is widely used in Japan and abroad, then global issues such as the exploitation of patients and their family members who are sacrificed to the detriment of the medical practitioners [22], could be addressed effectively.

However, in the past medical information providers have developed such guidelines as self-regulation by medical information providers [28]. We consider that it is difficult to expect compliance from those problematic clinics with the guidelines based on the spirit of self-regulation. In fact, although critical social views of stem cell therapies have grown more common, private clinics that offer unestablished stem cell therapies continue to disseminate easily accessible information on their websites. For this reason, a fundamental problem-solving approach that does not rely on self-regulation is also needed [14]. As an example of such an approach, one study suggested a promotion of the development of international certification standards for private-practice clinics offering stem cell therapies, as well as the joint creation of national policies in tandem with the policymakers of countries in which such clinics offer problematic noncovered treatments [14]. In order to create realistic standards, it is necessary to first investigate the actual situation of such clinics in various countries and their respective websites, and comprehensively analyze the obtained data.

### Conclusion

The present study examined websites, and does not investigate the actual conditions of regenerative medicine performed as treatment not covered by insurance. Moreover, not all Japanese clinics that provide such treatments were a target of study. However, in the present study, an exploratory analysis of the contents of website information confirmed the following points: (1) these sites have poor citation of scientific evidence, and (2) there were many websites that used marketing techniques such as name-dropping in order to solicit the attention of patients. Based on these findings, it has become clear that the website information provided by clinics that were a target of study paid insufficient considerations to patients and their family members and that there is a problem with the quality of the website information concerning cell therapies. It is important that more fact-finding studies be undertaken on a global scale so that a body of supervisory authorities can effectively implement an orchestrated regulatory approach toward private-practice clinics offering cell therapies across nations.

## Appendix

Multimedia Appendix 1 Scope of Application of the ASRM.

Multimedia Appendix 2 The 10 Quality Criteria of Minimum Standard from e-Health Code of Ethics 2.0.

Multimedia Appendix 3 Demographic Information of 24 Private-Practice Clinics.

## Abbreviations

ASRM: Act on Safety for Regenerative Medicine
JIMA: Japan Internet Medical Association
MHLW: Ministry of Health, Labour, and Welfare
