# Supplementary material for: Evaluating the Quality of Website Information of Private-Practice Clinics Offering Cell Therapies in Japan
Source: Interact J Med Res. 2016 May 24;5(2):e15. doi: 10.2196/ijmr.5479 (PMC4897299; doi:10.2196/ijmr.5479)
Supplement: Multimedia Appendix 1 [file ijmr_v5i2e15_app1.pdf]

Multimedia Appendix 1

Scope of Application of the ASRM

The definitions for regenerative medicine in the present study is based on the definitions found in the ASRM [11]: "The reconstruction, repair, or formation of the structure or function of the human body" or "medical treatments performed with the purpose of treating or preventing human diseases" and utilize "processed products of cells (Requirement 1)" and "Processed products" of cells refers to "the artificial proliferation and differentiation of cells and tissues, establishment of cell lines, pharmacotherapy or alterations of biological characteristics with the purpose of cell activation, combination of non-cellular components, or performing modifications through genetic engineering (Requirement 2)." However, this does not apply to blood transfusions and the widespread procedures of hematopoietic stem cell transplantation or assisted reproductive medicine. Therefore, the targets of analysis in the present study include not only stem cell treatments, but also all cell therapies used in regenerative medicine (e.g., cancer immunotherapy, platelet-rich plasma therapy).

Scope of Application of the ASRM

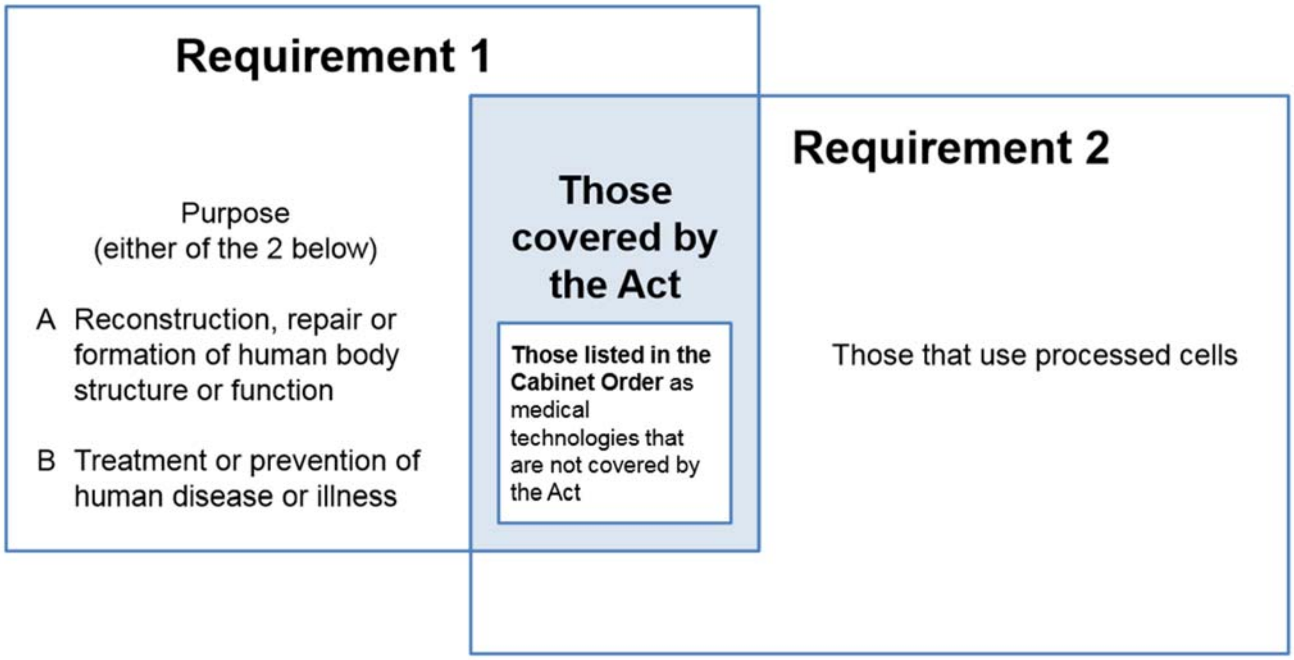

Source: http://www.mhlw.go.jp/english/policy/health-medical/medical-care/dl/150407-01.pdf
