# Supplementary material for: Evaluating the Quality of Website Information of Private-Practice Clinics Offering Cell Therapies in Japan
Source: Interact J Med Res. 2016 May 24;5(2):e15. doi: 10.2196/ijmr.5479 (PMC4897299; doi:10.2196/ijmr.5479)
Supplement: Multimedia Appendix 2 [file ijmr_v5i2e15_app2.pdf]

Multimedia Appendix 2

The 10 Quality Criteria of Minimum Standard from e-Health Code of Ethics 2.0

E1 Disclosure of information about the website operator

Unless the identity of the website operator (corporation or organization name, name of representative, address, telephone number, fax number, e-mail address, etc.) is made clear, the responsibility of information provision becomes unclear, increasing the risk of detriment to users.

E2 Disclosure of information about sponsorship

It is necessary that information concerning the sponsors be disclosed so that users can determine the impartiality of the displayed contents.

E3 Provision of contact center for further inquiries

Information concerning a contact center for further inquiries is necessary to ensure that users have an opportunity to know more about the information that is presented on the website and facilitate a response when a user suffers some sort of detriment due to said information.

E4 Clarifying intended recipients of content

In order for various end users (e.g., patients, portal site managers, librarians, medical specialists, government officials) to be able to access websites related to medical and health information and in order to ensure the expertise and readability of the contents, it is desirable to display the target recipient of said contents.

E5 Disclosure of the information concerning writing, production, and/or editorial supervision of conduct

The disclosure of information concerning the copyrights, producers, and editorial supervisors of the contents plays an important role in helping general users to understand highly technical information and determine the appropriateness of the information.

E6 Adherence to relevant laws and regulations

The code requirement for E6 from the Minimum Standard of e-health Code of Ethics says that online information provided by medical institutions should adhere to the

relevant laws and regulations or guidelines established by the Ministry of Health, Labour and Welfare (MHLW) in Japan. In our study, we referred to five negative lists contained in an official report from the MHLW [12] and used them for our evaluation. In Japan, clinic websites are currently not regarded as medical advertising, but there are two reasons why we selected this list. First, the lists contained specific examples of prohibited advertisements for medical institutions offering cell therapies, and the evaluation criteria of the list are clear and easy to use. Second, in recent years, there has been increasingly heated debate concerning the argument of regarding websites as a form of medical advertising [29-31]. In our study, we defined the coding frame of E6 as follows:

6-1) Prohibited advertising of names of the medical institutions (i.e. “Regenerative Medicine Clinic”)
The terms "regenerative medicine center" or "regenerative medicine clinic" were used, although such terms are prohibited by the Medical Care Act, Order for Enforcement of the Medical Care Act, and Ordinance for Enforcement of the Medical Care Act.

6-2) Prohibited advertising of names of hospital departments (i.e. “Department of Regenerative Medicine”)
The term "department of regenerative medicine" is used, although it is prohibited by the Medical Care Act, Order for Enforcement of the Medical Care Act, and Ordinance for Enforcement of the Medical Care Act.

6-3) Prohibited claim of specialization (i.e. “Certified Specialist of Regenerative Medicine")
The title "specialist in regenerative medicine," a specialist qualification that does not fall under the category of qualifications permitted to be advertised, is used.

6-4) Prohibited use of the term (i.e. "regenerative medicine") in the explanations of treatments
The advertisement contains pre- and post-operational photographs, which are considered to be improper.

6-5) Prohibited use of photos (i.e. claiming the effectiveness of a surgery by showing the pre- and post-operational photographs of patients)

The term "regenerative medicine," which is prohibited to be used in advertisements for medical treatments, was used.

E7 Notifications to the users of profit-oriented activities on the websites
Some profit-oriented websites provide information but are designed to generate profit through acting as an intermediary for the sale of products or specialized services. These profit-oriented activities must be specified to patients and their family members who use the websites.

E8 Displaying a pop-up message box that reminds the user of being transferred to the external websites when clicking the links to the external websites
Because the displaying of a pop-up message box that reminds the user of being transferred to the external websites when clicking the links to the external websites is an opportunity for the user to understand the nature of the site being viewed, pop-up displays are useful function for users to proactively search for information.

E9 Displaying handling of personal information
When the e-Health Code of Ethics 1.0 was first widely published by JIMA, many website creators could not differentiate displaying handling of personal information and displaying privacy policies. Thus, in order for website creators to be aware that their own websites deal with personal information at time of self-assessment, this item was made independent from the following item E10. In this item, we evaluated only the presence of displaying the handling of personal information.

E10 Displaying a privacy protection policy
The medical information-related sites not only handle the personal information of end users, but also often deal with online sensitive information concerning the health conditions and diseases of these users. In order to show the methods of managing accumulated personal information, purpose of use, confidentiality, information disclosure conditions, etc. are being appropriately handled, website operators bear the responsibility to display a privacy protection policy.
