# Supplementary material for: Evaluating the Quality of Website Information of Private-Practice Clinics Offering Cell Therapies in Japan
Source: Interact J Med Res. 2016 May 24;5(2):e15. doi: 10.2196/ijmr.5479 (PMC4897299; doi:10.2196/ijmr.5479)
Supplement: Multimedia Appendix 3 [file ijmr_v5i2e15_app3.pdf]

Multimedia Appendix 3
Demographic Information of 24 Private-Practice Clinics

Hospital ID	Location in Japan	Foreign Language Websites
1	Tôkyô	English, Chinese
2	Tôkyô	NA
3	Tôkyô	NA
4	Tôkyô	English
5	Tôkyô	English
6	Tôkyô	NA
7	Tôkyô	NA
8	Tôkyô	NA
9	Aichi	NA
10	Kyôto	NA
11	Tôkyô	NA
12	Tôkyô	English, Chinese
13	Fukuoka	NA
14	Tôkyô	NA
15	Ôsaka	NA
16	Tôkyô	NA
17	Tôkyô	NA
18	Tôkyô	Chinese
19	Tôkyô	NA
20	Tôkyô	NA
21	Ôsaka	NA
22	Tôkyô	NA
23	Aichi	NA
24	Fukuoka	NA
